# Man as an Object of Zoology

**Published:** 1888-01

**Authors:** S. W. Stiles

**Affiliations:** Georgia


### MAN AS AN OBJECT OF ZOOLOGY.

By S. W. Stiles, M.D., Ga.

[*Continued from page 443.*]

Differences equally striking are found in the cavity of the cranium of which the general capacity and particular forms depend entirely on the size and partial development of the brain. Hence our zoological survey of man will be greatly assisted by carefully examining genuine specimens of the skulls of different nations, which are easily prepared and preserved, may be conviently handled and surveyed, considered in various points of view, and compared to each other.

Such a comparison will show us that the form of the cranium differs no less than the color of the skin, or other characters ; and that one kind of structure runs, by gentle and invisible gradations, into another ; yet that there is, on the whole, an undeniable, nay a very remarkable constancy of character in the crania of different nations contributing very essentially to national peculiarities of form, and corresponding exactly to the features which characterize such nations. Hence, anatomists have attempted to lay down some scale of dimensions to which the various forms of the skull might be referred, and by means of which they might be reduced into certain classes. It often happens that crania of the most different nations, which differ in toto from each other on the whole, have same facial line. We are indebted to Blumenback for the completest body of information on this subject. This method of considering the bony head, he calls norma verticalis. The great expanse of the upper, and exterior part of the cranium, hiding the face, characterizes the Georgian. In the Etheopian, the narrow, slanting forehead allows the face to come into view, the cheeks and jaws are compressed laterally, and elongated in front. In the first, or white variety of men to which Blumenback has given the epithet Caucasian, including the ancient and modern inhabitants of Europe, the Western Asiatics, and the Northern Africans ; in a word, nearly all the inhabitants of the world, as known to the ancients. The upper and front parts of the skull are more developed than any other variety ; and their ample swell completely hides the face, when we survey the head according to the norma verticalis. The facial line must therefore be nearly vertical ; and the facial angle nearly a right angle. The face is comparatively small, and its outlines rounded without anything harsh or unpleasantly prominent. The cheek bones are small, and do not stand out, but descend in a nearly straight line from the external angular process of the frontal bone. The alreolar margin of the jaws is rounded, and the front teeth are perpendicular in both ; the chin is full and prominent. As a specimen, I refer to the skull of a Georgian woman, because it comes from a quarter near the supposed original seat of our race, and from a tribe celebrated for its personal beauty. From the elegance and symmetry of its formation, it may be regarded as the model of a female head ; and is certainly far preferable in this point of view to that of

"The bending statue which enchants the world."

The head of Venus, the Goddess of Love, is too small for an intellectual being, and the Goddess of Love is always represented as an idiot.

